# Relationship Between Within-Session Digital Motor Skill Acquisition and Alzheimer Disease Risk Factors Among the MindCrowd Cohort: Cross-Sectional Descriptive Study

**DOI:** 10.2196/67298

**Published:** 2025-04-24

**Authors:** Andrew Hooyman, Matt J Huentelman, Matt De Both, Lee Ryan, Kevin Duff, Sydney Y Schaefer

**Affiliations:** 1 Department of Physical Therapy, Chapman University, 9401 Jeronimo Rd, Irvine, CA, 92618, United States, 1 7146287208; 2 Division of Early Detection and Prevention, The Translational Genomics Research Institute, Phoenix, AZ, United States; 3 Department of Psychology, The University of Arizona, Tucson, AZ, United States; 4 Department of Neurology, Oregon Health and Science University, Portland, OR, United States; 5 School of Biological Health Systems Engineering, Arizona State University, Tempe, AZ, United States

**Keywords:** digital health technology, web-based assessment, aging, APOE, motor skills, sensitivity, risk factors, adults, older adults

## Abstract

**Background:**

Previous research has shown that in-lab motor skill acquisition (supervised by an experimenter) is sensitive to biomarkers of Alzheimer disease (AD). However, remote unsupervised screening of AD risk through a skill-based task via the web has the potential to sample a wider and more diverse pool of individuals at scale.

**Objective:**

The purpose of this study was to examine a web-based motor skill game (“Super G”) and its sensitivity to risk factors of AD (eg, age, sex, *APOE* ε4 carrier status, and verbal learning deficits).

**Methods:**

Emails were sent to 662 previous MindCrowd participants who had agreed to be contacted for future research and have their *APOE* ε4 carrier status recorded and those who were at least 45 years of age or older. Participants who chose to participate were redirected to the Super G site where they completed the Super G task using their personal computer remotely and unsupervised. Once completed, different Super G variables were derived. Linear and logistic multivariable regression was used to examine the relationship between available AD risk factors (age, sex, *APOE* ε4 carrier status, and verbal learning) and distinct Super G performance metrics.

**Results:**

Fifty-four participants (~8% response rate) from the MindCrowd web-based cohort (mean age of 62.39 years; 39 females; and 23 *APOE* ε4 carriers) completed 75 trials of Super G. Results show that Super G performance was significantly associated with each of the targeted risk factors. Specifically, slower Super G response time was associated with being an *APOE* ε4 carrier (odds ratio 0.12, 95% CI 0.02-0.44; *P*=.006), greater Super G time in target (TinT) was associated with being male (odds ratio 32.03, 95% CI 3.74-1192,61; *P*=.01), and lower Super G TinT was associated with greater age (*β* −3.97, 95% CI −6.64 to −1.30; *P*=.005). Furthermore, a sex-by-TinT interaction demonstrated a differential relationship between Super G TinT and verbal learning depending on sex (β_male:TinT_ 6.77, 95% CI 0.34-13.19; *P*=.04).

**Conclusions:**

This experiment demonstrated that this web-based game, Super G, has the potential to be a skill-based digital biomarker for screening of AD risk on a large scale with relatively limited resources.

## Introduction

Since the number of cases of Alzheimer disease (AD) is expected to double in the next 2 decades [1], there is an urgent need for widespread screening of older adults for their individual AD risk profile, which has implications for clinical care and research. Current options, such as positron emission tomography for measuring tau and beta amyloid pathology, tend to be expensive and invasive and require advanced imaging facilities [2]. Blood-based biomarkers may make screening for AD more affordable and less invasive, but their validity and standardization are still being established at this time [3,4]. Cognitive screening tests can indicate deficits, but these largely rely upon in-person administration from a trained clinician and may have reduced sensitivity in identifying individuals at high risk of AD in the earliest stages [5]. Thus, it is important to identify measures that are sensitive to AD risk and yet can also be delivered accurately, easily, and directly to patients and prospective participants in clinical trials and research studies.

Digital biomarkers have great potential to meet the need for accessible and remote testing of AD risk. Broadly speaking, digital biomarkers are measurable indicators of health or disease collected from a digital device or through digital means. Some digital biomarkers of AD include finger tapping [5,6], repeated cognitive assessment on a Wi-Fi–enabled device [7], recorded speech [8], and digital clock drawing [9], which have all been shown to be sensitive to cognitive impairment. These measures have also been associated with disease status, differentiating between cognitively intact versus mild cognitive impairment (MCI) and MCI versus dementia to some degree. However, these examples have overall low sensitivity to risk factors of AD, such as age [10], sex [11], apolipoprotein-E (*APOE*) ε4 carrier status [12,13], and brain amyloid [9]. Considering this, another potential digital biomarker of AD could be motor skill. Motor skill acquisition is the within-session improvement in a motor skill as a function of practice [14]. Previous in-lab studies have associated motor skill deficits with *APOE* carrier status, hippocampal atrophy, functional decline, and amyloid deposition among people diagnosed with amnestic MCI [15-18]. This task can also be collected remotely [19,20], which would allow for a wider and more diverse sample of individuals at potential risk for AD and can more easily facilitate longitudinal testing as desired.

This study developed a web-based tool for assessing motor skill performance called Super G [21], which can be reliably played unsupervised on the web [20] regardless of device type and without downloading any app. Specifically, the objective of this study was to examine whether Super G performance was individually related to known risk factors of AD (eg, age, sex, *APOE* ε4 carrier status, and verbal learning). Based on prior motor skill studies in AD, it was hypothesized that Super G performance would be negatively associated with each risk factor.

## Methods

### Study Design

This was a cross-sectional descriptive study that examined within-session performance characteristics from a web-based motor skill task (Super G) and their association with AD risk factors among adults recruited from the MindCrowd study.

### Participants

Participants were recruited in May of 2021 through MindCrowd, a web-based research study launched in 2013 to crowdsource demographic, medical history, lifestyle, and cognitive data to identify risk factors of AD [22,23]. Emails were sent through MindCrowd to a subset of 662 individuals who met our inclusion or exclusion criteria and were older than 45 years, who had previously provided a dried blood spot or saliva sample for *APOE* genotyping (see section “AD Risk Factors and Other Participant Characteristics From MindCrowd” for details) and had provided consent to be contacted for future studies. A hyperlink was included in the email that directed individuals to the Super G game website, on which participants digitally provided consent (approved by the Arizona State University institutional review board study no. 13081). Of the 662 individuals emailed, 54 participants (age: mean 62.39, SD 7.4 years; female: n=39) appropriately registered and completed all 75 trials of the game, equating to an 8.1% response rate. The mean (SD) time between MindCrowd data collection (specifically verbal learning, see section “AD Risk Factors and Other Participant Characteristics From MindCrowd” for details) and Super G data collection was 5.9 (1.4) years.

### Super G

The Super G game was developed in Unity 5.3.1 and is hosted on Hostinger. Thus, participants were not required to download an app or program to their device. Super G was developed as a gamified version of a seminal motor skill paradigm [24] and has been validated against the original version [21]. The goal of Super G is to help an astronaut explore as many planets as possible within the game’s solar system. There are 16 planets to visit but only 75 attempts to reach them all. Participants use the left and right arrow keys on their keyboard to move the astronaut onto a planet. However, the game uses a rate control mechanism that may not be immediately apparent to participants [21]. Specifically, pressing the right arrow key applies a constant positive force to move the astronaut toward the planet, while pressing the left arrow key applies an equal negative force away from the planet. Since the virtual environment lacks gravity or drag, any force applied will result in a constant velocity until a negative force is applied to slow it down. Thus, participants must learn to apply negative force at the right time and for the right duration to land the astronaut on each planet.

**Figure 1.**
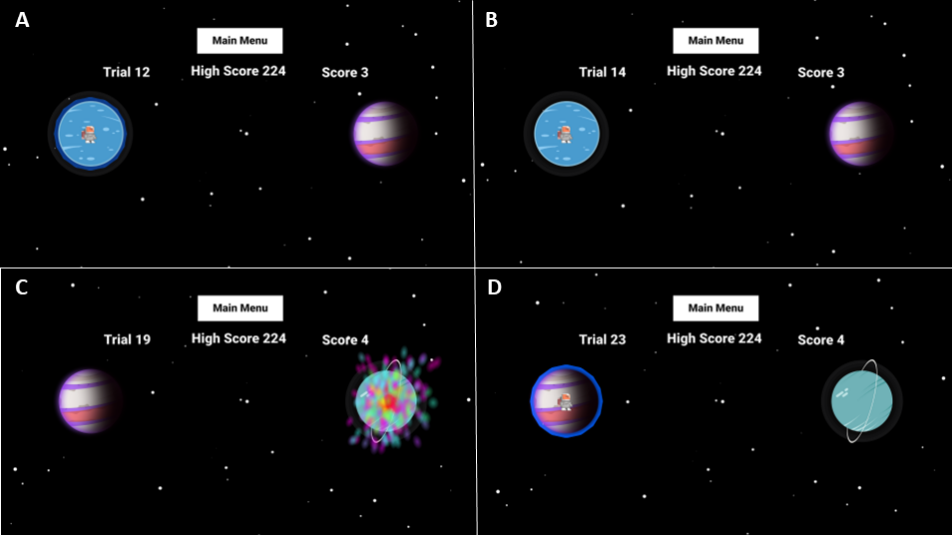
Each panel represents the different phases a participant may experience during Super G play. (A) The astronaut, Super G, spawns on the start planet at the beginning of each trial. There is a blue atmosphere around the planet that signals to the participant that Super G cannot leave yet. (B) Once the blue atmosphere disappears, then Super G can leave the start planet and attempt to land on the target planet. (C) If Super G successfully stays within the target planet boundary for 1 continuous second, then a reward tone plays and fireworks erupt from the planet. (D) Subsequent trials then render the previous target planet as the new start planet and a new target planet is put in place.

Each trial in Super G begins with the astronaut positioned on the left side of the screen on the initial start planet. The target planet is located on the opposite side of the screen to the right (Figure 1A). The trial lasts 4.5 seconds, but the astronaut cannot leave the start planet until 1.5 seconds have elapsed, as indicated by the disappearance of the blue atmosphere around the start planet (Figure 1B). If the astronaut leaves too early, the trial resets and the astronaut is returned to the initial position on the start planet. Once the blue atmosphere disappears, the participant has 3 seconds to land the astronaut on the target planet. To achieve a successful landing, the astronaut must stay within the boundary of the target planet for 1 continuous second. If successful, a reward tone plays, and fireworks erupt from the target planet (Figure 1C). After a successful landing, Super G repositions at the start position, and the previously landed planet becomes the start planet, with a new target planet appearing in its place (Figure 1D). In the event of a failed landing, the planets for the next trial remain the same, and astronaut reappears at the start position. All 75 trials are completed within a single session for a total session length of approximately 6 minutes.

Cursor position and key press data from each trial of Super G were collected at 100 Hz, along with high scores, time and date of each trial. From these data, four performance variables were calculated. First, scaling ratio (SR) represents the ratio of negative force (duration of left button press) applied to Super G over positive force (duration of right button press), where values equal to 1 indicate equal scaling of forces. Second, time of reversal (TR) is a measure of how well participants execute the timing of their movements by identifying when the left arrow key press occurs during the trial, where higher values are reversals that occur later in the trial. Successful trials require equal scaling of forces (ie, SR close to or equal to 1) and a reversal timed at the midway point of the trial, although we note that there can be trade-offs between the SR and TR to still allow for some degree of success in the task. Third, total time in the target (TinT) planet is the time that the cursor stayed within the target planet, with higher values representing better performance. Fourth, response time (RT) is a measure of how soon the cursor left the home planet after the disappearance of the start planet’s atmosphere, and lower values equate to a faster RT.

Although these performance metrics do strongly correlate with one another, each represents distinct phases of individual motor skill acquisition, as it is possible to have a fast RT with a low TinT if either timing of cursor reversal or scaling of forces is not well executed. Overall, average TinT across all 75 trials is the primary measure of performance since it directly represents the task goal and performance of the task as it relates to execution of SR and TR. Average RT across all 75 trials was considered as a secondary measure of performance because it represents the earlier stage of skill acquisition as participants first need to anticipate when to exit the start planet prior to execution of their acquired movement strategy. Average TinT and RT across the 75 trials are used as the Super G metrics of individual performance due to our prior work [21], which demonstrated that average TinT and RT well describe individual within-session change in performance and delayed retention.

### AD Risk Factors and Other Participant Characteristics From MindCrowd

Super G data were harmonized with MindCrowd through merging of hashed email addresses linked to MindCrowd data and used as the login for the Super G game. Super G variables were then merged with participant age, sex, level of education, verbal learning (measured via paired associates learning [PAL]) score [25], simple visual reaction time (svRT), and *APOE* genotype data, which were available in MindCrowd. The PAL is a verbal learning task that measures the ability to remember the associations between different word pairs. Specifically, participants are visually presented with 12 word pairs, with each word pair presented separately and at 2-second intervals. Participants were then presented with the first word of each pair and then used their keyboard to type the missing word. This procedure was repeated for 2 additional trials. The maximum score on the PAL is 36 (12 words across 3 trials). The svRT task measured the median reaction time across 6 trials, in which participants had to press any keyboard button as soon as a target stimulus appeared on their screen, with reaction time for each trial recorded in milliseconds. Median svRT was used due to the skewness of the distribution of reaction time in each participant’s individual data (skewness svRT median 1.2 vs skewness svRT mean 4.1). The svRT test was used in this study as a control variable only. Participants were classified as *APOE* ε4 carriers or noncarriers. Carriers were defined as individuals who had either 1 or 2 copies of the ε4 allele, and noncarriers were defined as individuals with 0 copies of the ε4 allele. This was based on prior genotyping from biospecimen collection via self-administered saliva or dried blood spot kits that were mailed to the participants by MindCrowd and then processed by the Translational Genomics Research Institute. Details regarding biospecimen collection and genotyping can be found here [26]. Complete visualization of *APOE* ε4 carrier status with svRT, PAL, and age can be viewed in Figure S1 in Multimedia Appendix 1.

### Statistical Analysis

Wilcoxon rank sum tests were used due to a difference in sample sizes between *APOE* ε4 carriers (n=23) and noncarriers (n=31), since uneven sample sizes may lead to inaccurate and disproportionate estimates of variance of each group and violate assumptions of parametric testing. This determined whether there were differences in age, PAL score, svRT, and Super G performance between the 2 groups. Chi-square tests were used to determine whether there was a difference in proportion based on sex, race, education, and ethnicity between carriers and noncarriers. To provide broader context of the verbal learning (PAL) and reaction time (svRT) of this sample, percentile scores were calculated for each participant adjusted for their age, sex, and level of education. This allowed for better contextualization of the relative performance on each measure between carriers and noncarriers relative to the entire MindCrowd cohort (ie, if participants in this study are under- or overperformers compared with what would be expected to a random sample across the entire cohort).

Separate linear and logistic multivariable regression analyses, depending on outcome variable type, were used to test the relationship between Super G performance and the dependent variables, that is, AD risk factors. Specifically, different models were constructed to identify which Super performance metrics are related to specific AD risk factors, while controlling for possible confounding effects of the other AD risk factors and participant characteristics. Multivariable logistic regression was used for the dependent variable of *APOE* ε4 carrier status (where carriers were coded as “true” and noncarriers as “false”), along with the primary and secondary measures of Super G performance (TinT and RT), and control variables of age, sex, PAL score, hour of day Super G played, level of education, and svRT. This approach controlled for the other factors and was repeated with sex as the dependent variable (whereby male was coded as “true” and female as “false”) while switching *APOE* ε4 carrier status to a control variable. Multivariable regression was used when the dependent variable was PAL score, with a primary independent variable of mean Super G performance (TinT, RT, SR, or TR), and control variables of age, sex, *APOE* ε4 carrier status, hour of day Super G played, level of education, and svRT. The same approach was repeated with age as the dependent variable while switching PAL score to a control variable. In addition, to control for the potential delay between initial PAL scores and Super G measurement, PAL-adjusted scores were also generated and analyzed. Individual PAL scores were adjusted based on the amount of time between their PAL and Super G measurement, given previously reported expected decline in PAL based on age reported by Talboom and colleagues [26]. This resulted in an expected 0.2-point decline in PAL for every year between the initial PAL and current Super G measurement. The formulation of each model can be viewed in the following equations:

1. *APOE* ε4 carrier status ~ Super G Performance (TinT or RT) + age +sex + hour played + education +svRT + PAL (Logistic regression)
2. Sex ~ Super G Performance (TinT or RT) + age + hour played + education + svRT + PAL + *APOE* ε4 carrier status (Logistic regression)
3. Age ~ Super G Performance (TinT or RT) + sex + hour played + education + svRT + PAL + *APOE* ε4 carrier status (Linear regression)
4. PAL ~ Super G Performance (TinT or RT) + age + sex + hour played + education + svRT + *APOE* ε4 carrier status (Linear regression)
5. PAL adjusted ~ Super G Performance (TinT or RT) + age + sex + hour played + education + svRT + *APOE* ε4 carrier status (Linear regression)

Participants could play Super G at any time throughout the day, the variable of hour played (measured with a 24-hour clock rather than a 12-hour clock) was transformed using a cosine function to ensure that adjacent hours 0 and 23 were close together. This is visualized in Figure S2 in Multimedia Appendix 1. All numeric variables were standardized (age, svRT, PAL, hour played, and Super G Performance) to be centered at their respective mean and divided by their respective SD. Thus, a 1-unit change in the results of these variables with respect to all reported odds ratios (ORs) and beta coefficients represents a 1 SD change in either the outcome or the performance metric. This allowed for better relative comparison between all independent variables within each model. To detect multicollinearity, the variance inflation factor was calculated, and any variable with a variance inflation factor of >5 was removed. Outliers were identified using Cook’s distance and removed if their distance was >1.

### Ethical Considerations

The study protocol was reviewed and approved by the office of Research Integrity and Assurance at Arizona State University (approval number STUDY00013081). To participate, participants needed to provide digital informed consent. All data collected from this experiment were deidentified for privacy and confidentiality. Participants were not compensated for their time in this study.

## Results

### Participant Characteristics

Overall, there were 23 *APOE* ε4 carriers (20 heterozygotes and 3 homozygotes) and 31 noncarriers. Between-group comparisons (carriers vs noncarriers) using the Wilcoxon rank sum test demonstrated that groups did differ by age (W=471; *P*=.04), with noncarriers being an average of 4.3 years older than carriers (carriers=59.9 years, noncarriers=64.2 years). For all other control variables, there were no observed group differences (Table 1).

**Table 1.** Genetic, demographic, cognitive, motor, and Super G performance data between *APOE* ε4 carries and noncarriers.

	*APOE* ε4 carriers	*APOE* ε4 noncarriers	Test statistic (*df*)	*P* value
Participants, n	23	31	—^a^	—
*APOE* alleles (X/X), n			—	—
2/2	—	0		
2/3	—	2		
3/3	—	29		
2/4	4	—		
3/4	16	—		
4/4	3	—		
Age (years), mean (SD)	59.9 (7.4)	64.2 (6.9)	*W*=471 (—)	.045^b^
Sex (male/female)	6/17	9/22	*χ*^2^=0 (1)	>.99
Race			*χ*^2^=0 (1)	>.99
White	23	31		
Ethnicity			*χ*^2^=0 (1)	>.99
Not Latinx	23	31		
Education, n			*χ*^2^=2.5 (3)	.47
High school diploma	1	0		
Some college	5	4		
Four-year degree	9	12		
Postgraduate degree	8	15		
PAL^c^ score, mean (SD)	23.4 (8.4), 66th percentile	19.2 (8.5), 54th percentile	*W*=254 (—)	.07
median svRT^d^ (ms), mean (SD)	389.7 (75), 71st percentile	418 (79), 65th percentile	*W*=451 (—)	.10
Hour of day played, mean (SD)^e^	13.1 (4.6)	14.9 (4.1)	*W*=441 (—)	.14
Hour of day played (cosine), mean (SD)	0.52 (0.6)	.45 (6)	*W*=394 (—)	.49
Super G time in target (ms), mean (SD)	653.9 (436.6)	469.4 (287.9)	*W*=279 (—)	.18
Super G response time (ms), mean (SD)	1614.8 (305.9)	1874.5 (307.4)	*W*=502 (—)	.01^b^
Super G time of reversal (ms), mean (SD)	2000.6 (592.4)	2060.3 (836.4)	*W*=432 (—)	.19
Super G scaling ratio, mean (SD)	0.66 (0.3)	0.64 (0.3)	*W*=343 (—)	.82

a N/A: not applicable.

b Statistical significance (*P*<.05).

c PAL: paired associates learning.

d svRT: simple visual reaction time.

e Based on 24-hour clock.

### Relationship Between Super G Performance and AD Risk Factors

**Figure 2.**
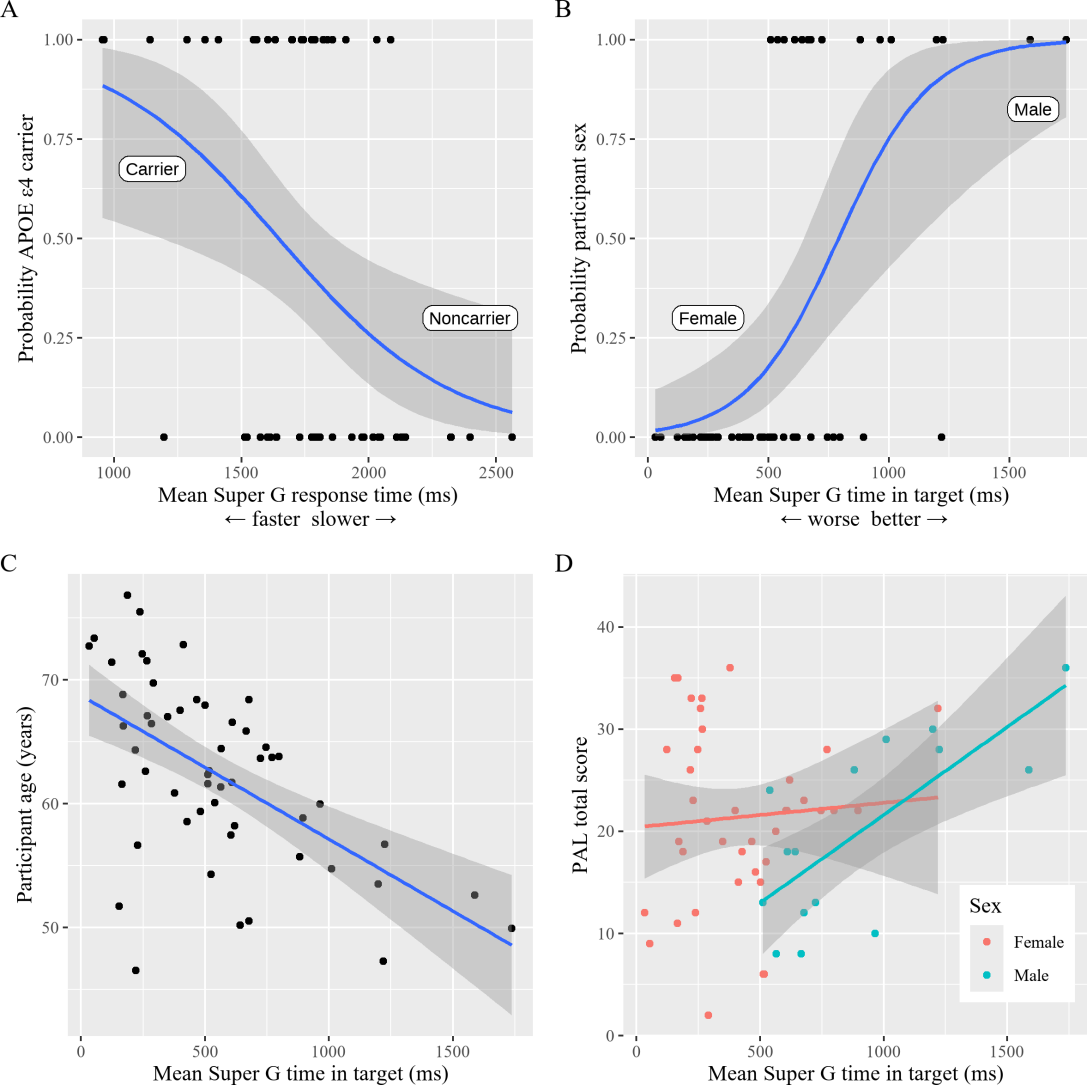
(A) Mean Super G response time (ie, time at which Super G exits the start planet once the blue atmosphere disappears) between *APOE* ε4 noncarriers and carriers. Y-axis represents the probability a participant is classified as a carrier or noncarrier based on their mean response time. The blue line represents the binomial relationship between response time and carrier status with faster response time more associated with being a carrier and slower response time more associated with being a noncarrier. The gray ribbon represents the 95% CI of the estimated probability of binomial relationship. (B) Mean Super G time in target between female and male participants. Y-axis represents the probability a participant is classified as a male or female based on their mean time in target. The blue line represents the binomial relationship between time in target and sex with better time in target associated with being a male and worse time in target associated with being a female. The gray ribbon represents the 95% CI of the estimated probability of binomial relationship. (C) Linear relationship between participant age in years and their mean Super G time in target. The blue line represents the least squares line fitted between the variable and the gray ribbon about the line represents the 95% CI. (D)The relationship between mean Super G time in target to individual PAL total score stratified by sex (male in blue and female in pink). Given known sex differences between males and females on PAL and observed sex differences on mean Super time in target, such a stratification by sex was necessary to control for potential confounding of sex on the Super G to PAL relationship. The blue line represents the line of least squares for males and the pink line represents the line of least squares for females. The gray ribbon about each line represents the 95% CI. PAL: paired associates learning.

Within the *APOE* ε4 carrier status logistic regression (model 1) there was a significant association between *APOE* ε4 carrier status with Super G RT (OR 0.12, 95% CI 0.02-0.44; *P*=.006) (Figure 2A). Thus, a participant with a Super G RT that was 1 SD below the mean would have an 88% increase in odds of being an *APOE* ε4 noncarrier than carrier. Visual comparison between all Super G performance metrics and *APOE* ε4 can be visualized in Figure S3 in Multimedia Appendix 1. All other control variables, including PAL (*P*=.62) and svRT (*P*=.29), were not significantly related to *APOE* ε4 carrier status. Within the participant age linear regression (model 3), Super G TinT was significantly associated with age (*β* −3.97, 95% CI −6.64 to −1.30; *P*=.005) (Figure 2C) and within the participant sex logistic regression (model 2), TinT was also associated with sex (OR 32.03, 95% CI 3.74-1192.61; *P*=.01) (Figure 2B), with lower TinT values (poorer performance) associated with being older and being female, respectively. This is consistent with our earlier work in other cohorts [27]. Visual comparison between all Super G performance metrics and sex can be visualized in Figure S4 in Multimedia Appendix 1.

Given that previous research in MindCrowd has demonstrated a main effect of sex on verbal learning (as measured with the PAL test) [26,28], and that TinT is also strongly linked with participant sex (Figure 2B), within the PAL linear regression (model 4), a sex-by-TinT interaction was also included to best model PAL [26]. Results from the PAL model demonstrated that age was associated with PAL (*β* −3.37, 95% CI −6.34 to −0.4; *P*=.03), indicating that older age was associated with lower PAL scores. There was also a main effect of participant sex (β_Male_ −7.73, 95% CI −14.98 to −0.45; *P*=.04). This result indicates that male participants scored an average of 7 points lower on the PAL than females. There was a significant sex-by-TinT interaction (β_Male:TinT_ 6.77, 95% CI 0.34-13.19; *P*=.04). This result indicates that males with a mean TinT 1 SD greater than the group mean are associated with an increase of 6.77 points on the PAL compared with females with the same TinT performance (Figure 2D). Furthermore, in the PAL adjusted regression (model 5), where PAL scores are modified based on the time between PAL and Super G measures, the results are nearly identical to the raw scores (β_age_ −3.41, 95% CI −6.38 to −0.44; *P*=.03; β_Male_ −7.57, 95% CI −14.81 to −0.33; *P*=.04; β_Male:TinT_ 6.72, 95% CI 0.31-13.14; *P*=.04; Figure S5 in Multimedia Appendix 1). The results of this adjusted analysis provide evidence that the delay between PAL and Super G measures does not significantly impact the observed relationship between PAL and TinT given participant sex.

## Discussion

This study tested whether motor skill acquisition, as assessed by the Super G task, was sensitive to known risk factors of AD, namely age, sex, *APOE* ε4 carrier status, and verbal learning. Results showed that better Super G performance was directly and independently associated with each of these risk factors, suggesting that Super G may be a sensitive digital biomarker of AD risk that can be remotely collected. Interestingly, TinT and RT were related to different AD risk factors (sex, age, and verbal learning vs *APOE* ε4 carrier status, respectively), likely reflecting how different aspects of motor skill map onto different AD risk factors. Our prior work showed that TinT is the product of optimal execution of SR and TR and best characterizes overall skill compared with the other Super G variables [21], and it was related to age, sex, and verbal learning. However, RT is a performance characteristic of early skill acquisition, as it measures an individual’s ability to anticipate when to initialize movement at the beginning of the trial, which was related to *APOE* ε4 carrier status. Although *APOE* ε4 carriers unexpectedly outperformed noncarriers in terms of early skill acquisition (ie, better RT performance), this opens up the possibility of using Super G as a prognostic enrichment strategy for enriching AD-focused cohorts with ε4 carriers [29].

The observed *APOE* ε4 benefit in this study is contrary to previous work where *APOE* ε4 carrier status leads to worse performance on memory tests in carriers compared with noncarriers [30-32]. However, the role *APOE* ε4 in aging independent of AD pathology may be significant [33], as a small but growing body of evidence in both cognitively unimpaired humans and rodents shows that visual working memory and learning is better among *APOE* ε4 carriers than noncarriers [34-36]. Although *APOE* ε4 is the primary genetic risk factor for AD [13], evidence suggests a possible benefit, or compensatory behavior [37], of learning at an earlier age while leading to impairments in later life [38,39]. Previous research has shown that cognitively unimpaired individuals who are *APOE* ε4 carriers have greater gray matter volume in frontal regions, can better allocate cognitive control, and possess better visual working memory and learning than *APOE* ε4 noncarriers [34,40,41]. Furthermore, task-based neuroimaging studies have associated better performance on visual working memory tasks among carriers with greater activation in frontal and parietal regions [34,36,42]. This leads to the hypothesis that increased frontal activation is a compensatory mechanism that modifies behavior in AD, given that frontal brain regions are relatively spared in AD [43,44]. Although these data do not provide direct support for the *APOE* ε4 compensatory mechanisms, it may be plausible that Super G may be able to measure a suspected *APOE* ε4 benefit. This interpretation is further supported by the fact that in-lab motor skill studies have associated greater skill with higher white matter integrity of frontoparietal tracts [45,46] in cognitively unimpaired older adults, which could serve as a candidate neural substrate for such a compensatory mechanism as proposed previously, but further study would be needed to confirm.

Prior research that has investigated sex differences in motor skill learning and performance has demonstrated a preferential advantage for males compared with females [47]. Similarly, this study revealed that males tended to perform better than females on Super G. The estimated effect size of mean time in target between males and females was very large, with Cohen *d*=1.66. This result is consistent with previous research using Super G, which also found large sex differences in performance [27]. Several factors may explain the observed sex differences in Super G performance. One possible biological explanation [48] may be due to early tau deposition which has been shown to be elevated in females compared with males, consistent with the higher risk for developing AD among females. Thus, a remote and unsupervised motor skill task that is sensitive to sex differences may aid in the detection of sex-specific changes in behavior due to disease in a scalable way. Moreover, there was an interaction between sex, verbal learning, and Super G performance, which suggests that motor skill in males (ie, Super G performance) may be linked to their verbal learning to a greater degree than in females. Larger sample sizes are needed to examine the interactions between sex and other behavioral variables in the context of AD [49,50].

Several limitations to this study should be acknowledged. First, the mean time between when the PAL and Super G was 5 years. Although this is a substantial delay between 2 variables of interest, there was no correlation between PAL score and this intertest interval. Furthermore, when PAL was adjusted for this delay, there was no change in the reported relationship between Super G and PAL regardless of whether raw or adjusted PAL scores were used as the dependent variable (Figure S5 in Multimedia Appendix 1). Thus, the reported result between PAL and Super G appears robust, even with such a delay between measures. Second, the study sample was all non-Hispanic White and highly educated (>80% with at least a college degree), preventing any analysis of interactions between race or ethnicity and *APOE* ε4. Analyses that consider the interaction between race and carrier status are important, given that the link between the *APOE* ε4 allele and the AD is weaker among Black/African American and Hispanic/Latino individuals despite their increased risk of developing AD overall [12,51,52]. In addition, the study sample size was relatively limited due to our required inclusion criteria, particularly the existence of *APOE* genotyping data. As such, future Super G research will recruit larger and more diverse samples. We plan to expand the size of this work with future collaborations, and it is important to note that the MindCrowd cohort itself has increased in size, in racial and ethnic diversity, and in the number of individuals in the cohort who have *APOE* genotyping data, suggesting a path to addressing this limitation in the future. Third, we did not collect participant data on previous video game experience, which may be a contributing factor to the observed relationship between Super G performance and sex [53]. Fourth, there is no defined minimal clinically important difference or clinical cutoff score for the PAL, making it difficult to determine the meaningfulness of the observed relationship between PAL and Super G performance. For example, it cannot be determined whether the predicted change in PAL of 6.5 points among males, in relation to TinT performance, is representative of a meaningful increase or decrease in verbal learning. Finally, with only an 8.1% response rate in this study (54 participants completed all trials of Super G out of the 662 contacted), there may be limited generalizability to the broader MindCrowd cohort or the general public. However, this response rate is similar to other web-based AD-focused cohorts such as the Alzheimer’s Prevention Trials Webstudy [54], although the participants in this study were not paid to participate (in contrast to the Alzheimer’s Prevention Trials Webstudy), were not actively seeking care, and were emailed only once during the recruitment process. It is noted that these are the factors that could influence participation rates. Despite these limitations, this study establishes the proof of concept that Super G may be a feasible skill-based digital biomarker of individual AD risk.

## Supplementary material

10.2196/67298 Multimedia Appendix 1 Supplemental figures for between-group comparison across covariates and outcome measures contrasted with different Super G outcomes.

## Abbreviations

AD: Alzheimer disease
MCI: mild cognitive impairment
OR: odds ratio
PAL: paired associates learning
RT: response time
SR: scaling ratio
svRT: simple visual reaction time
TinT: time in the target
TR: time of reversal

## References

[1] Matthews KA, Xu W, Gaglioti AH (2019). Racial and ethnic estimates of Alzheimer’s disease and related dementias in the United States (2015-2060) in adults aged ≥65 years. Alzheimers Dement.

[2] Klunk WE (2011). Amyloid imaging as a biomarker for cerebral β-amyloidosis and risk prediction for Alzheimer dementia. Neurobiol Aging.

[3] Nakamura A, Kaneko N, Villemagne VL (2018). High performance plasma amyloid-β biomarkers for Alzheimer’s disease. Nature New Biol.

[4] Chong MS, Sahadevan S (2005). Preclinical Alzheimer’s disease: diagnosis and prediction of progression. Lancet Neurol.

[5] Dagum P (2018). Digital biomarkers of cognitive function. NPJ Digit Med.

[6] Omberg L, Chaibub Neto E, Perumal TM (2022). Remote smartphone monitoring of Parkinson’s disease and individual response to therapy. Nat Biotechnol.

[7] Papp KV, Jutten RJ, Soberanes D (2024). Early detection of amyloid-related changes in memory among cognitively unimpaired older adults with daily digital testing. Ann Neurol.

[8] Mueller KD, Van Hulle CA, Koscik RL (2021). Amyloid beta associations with connected speech in cognitively unimpaired adults. Alzheimers Dement (Amst).

[9] Rentz DM, Papp KV, Mayblyum DV (2021). Association of digital clock drawing with PET amyloid and tau pathology in normal older adults. Neurology (ECronicon).

[10] Gurland BJ, Wilder DE, Lantigua R (1999). Rates of dementia in three ethnoracial groups. Int J Geriatr Psychiatry.

[11] Podcasy JL, Epperson CN (2016). Considering sex and gender in Alzheimer disease and other dementias. Dialogues Clin Neurosci.

[12] Tang MX, Stern Y, Marder K (1998). The APOE-epsilon4 allele and the risk of Alzheimer disease among African Americans, whites, and Hispanics. JAMA.

[13] Liu CC, Liu CC, Kanekiyo T, Xu H, Bu G (2013). Apolipoprotein E and Alzheimer disease: risk, mechanisms and therapy. Nat Rev Neurol.

[14] Schmidt R, Lee T, Winstein C, Wulf G, Zelaznik H (2019). Motor Control and Learning: A Behavioral Emphasis.

[15] Rogojin A, Gorbet DJ, Hawkins KM, Sergio LE (2019). Cognitive-motor integration performance is affected by sex, APOE status, and family history of dementia. J Alzheimers Dis.

[16] Schaefer SY, Malek-Ahmadi M, Hooyman A, King JB, Duff K (2022). Association between motor task performance and hippocampal atrophy across cognitively unimpaired, amnestic mild cognitive impairment, and Alzheimer’s disease individuals. J Alzheimers Dis.

[17] Schaefer SY, Hooyman A, Duff K (2020). Using a timed motor task to predict one-year functional decline in amnestic mild cognitive impairment. J Alzheimers Dis.

[18] Schaefer SY, Duff K, Hooyman A, Hoffman JM (2022). Improving prediction of amyloid deposition in mild cognitive impairment with a timed motor task. Am J Alzheimers Dis Other Demen.

[19] Tsay JS, Lee A, Ivry RB, Avraham G (2021). Moving outside the lab: the viability of conducting sensorimotor learning studies online. Neuron Behav Data Anal Theory.

[20] Hooyman A, Huentelman MJ, De Both M, Ryan L, Schaefer SY (2023). Establishing the validity and reliability of an online motor learning game: applications for Alzheimer’s disease research within MindCrowd. Games Health J.

[21] Hooyman A, Gordon J, Winstein C (2021). Unique behavioral strategies in visuomotor learning: hope for the non-learner. Hum Mov Sci.

[22] Talboom JS, De Both MD, Naymik MA (2021). Two separate, large cohorts reveal potential modifiers of age-associated variation in visual reaction time performance. NPJ Aging Mech Dis.

[23] Lewis CR, Talboom JS, De Both MD (2021). Smoking is associated with impaired verbal learning and memory performance in women more than men. Sci Rep.

[24] Brooks V, Hilperath F, Brooks M, Ross HG, Freund HJ (1995). Learning “what” and “how” in a human motor task. Learn Mem.

[25] Pike KE, Rowe CC, Moss SA, Savage G (2008). Memory profiling with paired associate learning in Alzheimer’s disease, mild cognitive impairment, and healthy aging. Neuropsychology.

[26] Talboom JS, Håberg A, De Both MD (2019). Family history of Alzheimer’s disease alters cognition and is modified by medical and genetic factors. Elife.

[27] Hooyman A, Schaefer SY (2023). Age and sex effects on Super G performance are consistent across internet devices. Int J Serious Games.

[28] Kaushanskaya M, Marian V, Yoo J (2011). Gender differences in adult word learning. Acta Psychol (Amst).

[29] Cummings J, Apostolova L, Rabinovici GD (2023). Lecanemab: appropriate use recommendations. J Prev Alzheimers Dis.

[30] Caselli RJ, Reiman EM, Osborne D (2004). Longitudinal changes in cognition and behavior in asymptomatic carriers of the APOE e4 allele. Neurology (ECronicon).

[31] Marioni RE, Campbell A, Scotland G, Hayward C, Porteous DJ, Deary IJ (2016). Differential effects of the APOE e4 allele on different domains of cognitive ability across the life-course. Eur J Hum Genet.

[32] Evans SL, Dowell NG, Prowse F, Tabet N, King SL, Rusted JM (2020). Mid age APOE ε4 carriers show memory-related functional differences and disrupted structure-function relationships in hippocampal regions. Sci Rep.

[33] Palmer JM, Huentelman M, Ryan L (2023). More than just risk for Alzheimer’s disease: APOE ε4’s impact on the aging brain. Trends Neurosci.

[34] Lu K, Nicholas JM, Pertzov Y (2021). Dissociable effects of APOE-ε4 and β-amyloid pathology on visual working memory. Nat Aging.

[35] McLean JW, Bhattrai A, Vitali F, Raikes AC, Wiegand JP, Brinton RD (2022). Contributions of sex and genotype to exploratory behavior differences in an aged humanized APOE mouse model of late-onset Alzheimer’s disease. Learn Mem.

[36] Scheller E, Peter J, Schumacher LV (2017). APOE moderates compensatory recruitment of neuronal resources during working memory processing in healthy older adults. Neurobiol Aging.

[37] Filbey FM, Chen G, Sunderland T, Cohen RM (2010). Failing compensatory mechanisms during working memory in older apolipoprotein E-epsilon4 healthy adults. Brain Imaging Behav.

[38] Iacono D, Feltis GC (2019). Impact of apolipoprotein E gene polymorphism during normal and pathological conditions of the brain across the lifespan. Aging (Albany NY).

[39] Palmer JM, Grilli MD, Lawrence AV, Ryan L (2023). The impact of context on pattern separation for objects among younger and older apolipoprotein ϵ4 carriers and noncarriers. J Int Neuropsychol Soc.

[40] Dean DC, Jerskey BA, Chen K (2014). Brain differences in infants at differential genetic risk for late-onset Alzheimer disease. JAMA Neurol.

[41] Zink N, Bensmann W, Arning L, Beste C, Stock AK (2019). Apolipoprotein ε4 is associated with better cognitive control allocation in healthy young adults. Neuroimage.

[42] Wishart HA, Saykin AJ, Rabin LA (2006). Increased brain activation during working memory in cognitively intact adults with the APOE epsilon4 allele. Am J Psychiatry.

[43] Tuminello ER, Han SD (2011). The apolipoprotein E antagonistic pleiotropy hypothesis: review and recommendations. Int J Alzheimers Dis.

[44] Han SD, Bondi MW (2008). Revision of the apolipoprotein E compensatory mechanism recruitment hypothesis. Alzheimers Dement.

[45] Lingo VanGilder J, Bergamino M, Hooyman A (2022). Using whole-brain diffusion tensor analysis to evaluate white matter structural correlates of delayed visuospatial memory and one-week motor skill retention in nondemented older adults: a preliminary study. PLoS One.

[46] Regan E, Fridriksson J, Schaefer SY (2021). Neural correlates of within-session practice effects in mild motor impairment after stroke: a preliminary investigation. Exp Brain Res.

[47] Moreno-Briseño P, Díaz R, Campos-Romo A, Fernandez-Ruiz J (2010). Sex-related differences in motor learning and performance. Behav Brain Funct.

[48] Buckley RF, Mormino EC, Rabin JS (2019). Sex differences in the association of global amyloid and regional tau deposition measured by positron emission tomography in clinically normal older adults. JAMA Neurol.

[49] Caselli RJ, Langlais BT, Dueck AC (2020). Neuropsychological decline up to 20 years before incident mild cognitive impairment. Alzheimers Dement.

[50] Savignac C, Villeneuve S, Badhwar A (2022). APOE alleles are associated with sex-specific structural differences in brain regions affected in Alzheimer’s disease and related dementia. PLoS Biol.

[51] Farrer LA, Cupples LA, Haines JL (1997). Effects of age, sex, and ethnicity on the association between apolipoprotein E genotype and Alzheimer disease. A meta-analysis. APOE and Alzheimer Disease Meta Analysis Consortium. JAMA.

[52] Harwood DG, Ownby RL (2000). Ethnicity and dementia. Curr Psychiatry Rep.

[53] Terlecki M, Brown J, Harner-Steciw L (2011). Sex differences and similarities in video game experience, preferences, and self-efficacy: implications for the gaming industry. Curr Psychol.

[54] Walter S, Clanton TB, Langford OG (2020). Recruitment into the Alzheimer Prevention Trials (APT) Webstudy for a Trial-Ready Cohort for Preclinical and Prodromal Alzheimer’s Disease (TRC-PAD). J Prev Alzheimers Dis.

[55] Hooyman A Super G and AD risk factors in Mindcrowd. OSF.

