# Supplementary material for: Relationship Between Within-Session Digital Motor Skill Acquisition and Alzheimer Disease Risk Factors Among the MindCrowd Cohort: Cross-Sectional Descriptive Study
Source: JMIR Aging. 2025 Apr 24;8:e67298. doi: 10.2196/67298 (PMC12045524; doi:10.2196/67298)
Supplement: Multimedia Appendix 1 [file aging-v8-e67298-s001.pdf]

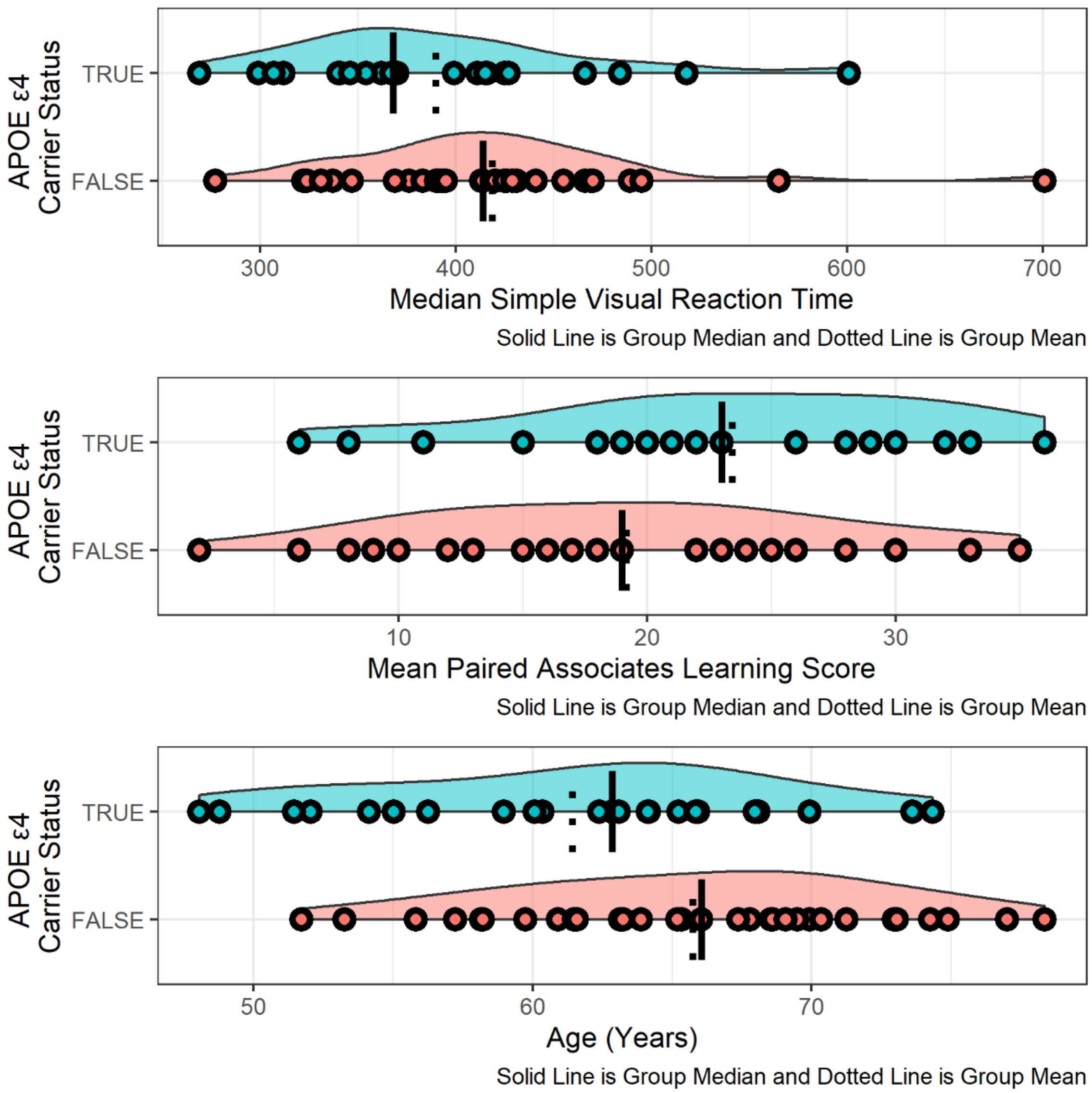

Fig 1S. Simple visual reaction time between APOE ε4 carrier status groups. Blue density distribution represents APOE ε4 carriers and pink represents non-carriers. Solid vertical line represents the group median and the dotted line represents the group mean.

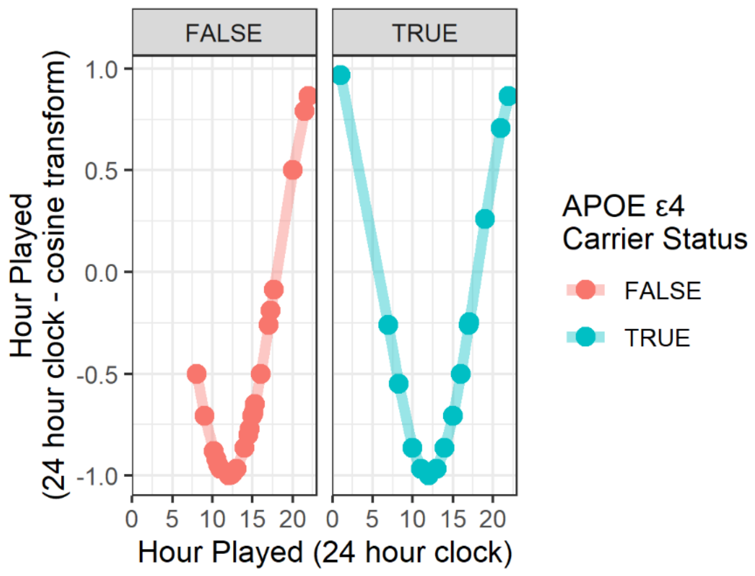

Fig 2S. Hour Super G played between APOE ε4 carrier status groups. Hour Super G played on a 24 hour clock displayed on the x axis and its cosine transformed value on the y-axis.

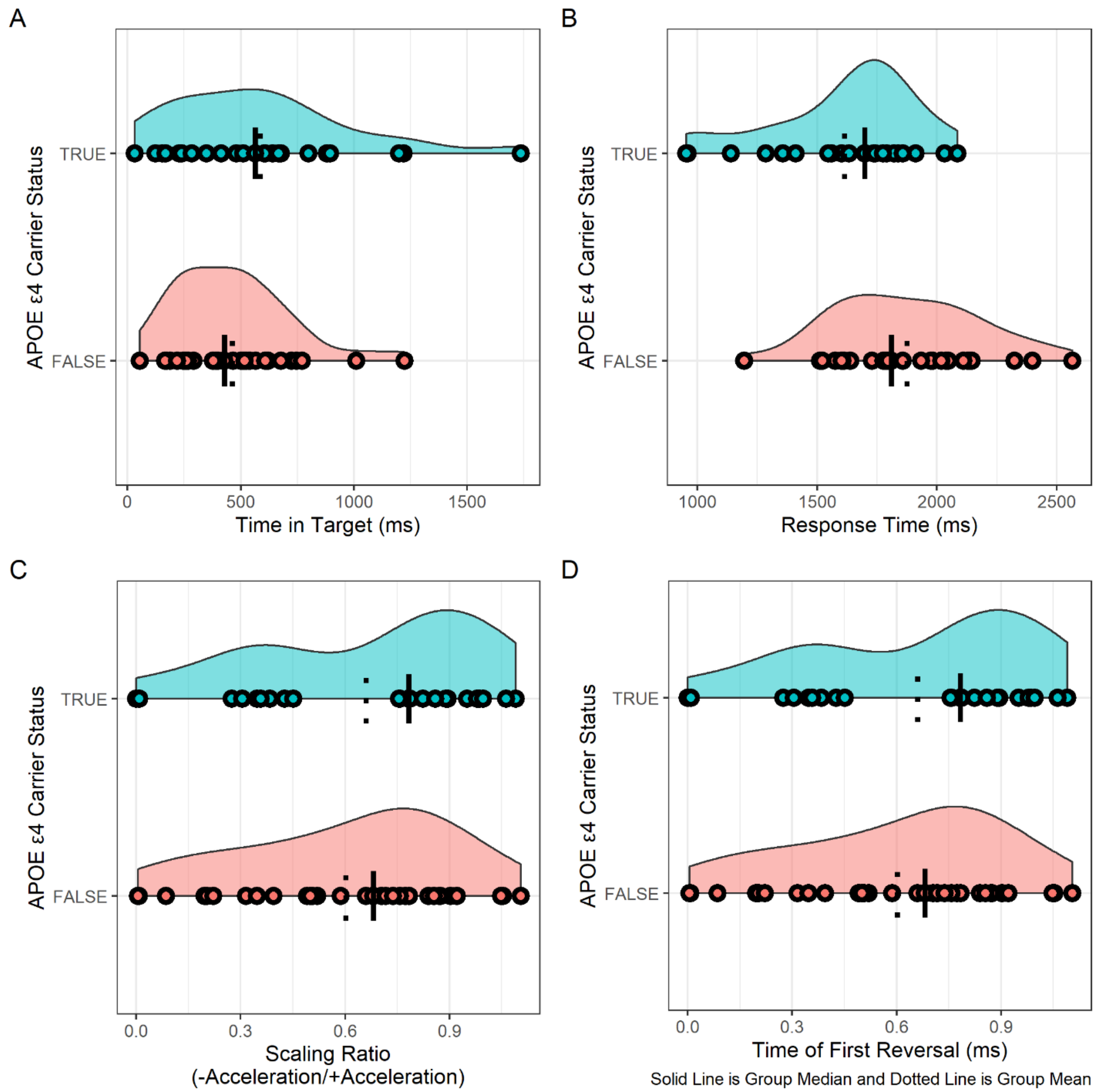

Fig 3S. Differences in APOE ε4 carriers across the different Super G performance variables. Blue density distribution represents APOE ε4 carriers and pink represents non-carriers. Solid vertical line represents the group median and the dotted line represents the group mean.

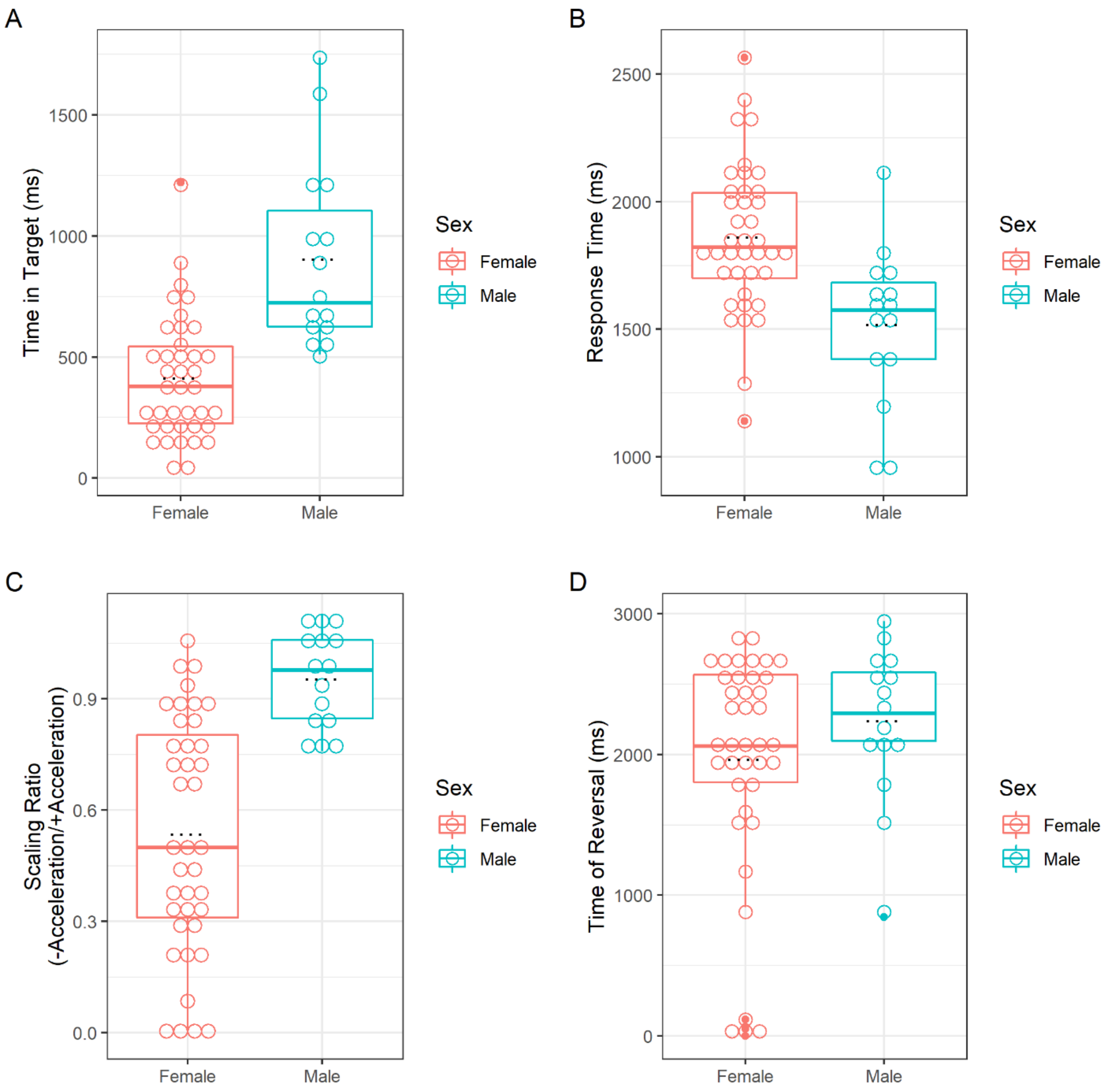

Fig 4S. Differences between male and females across the different Super G performance variables. Blue boxplots represent males and pink boxplot represent females.

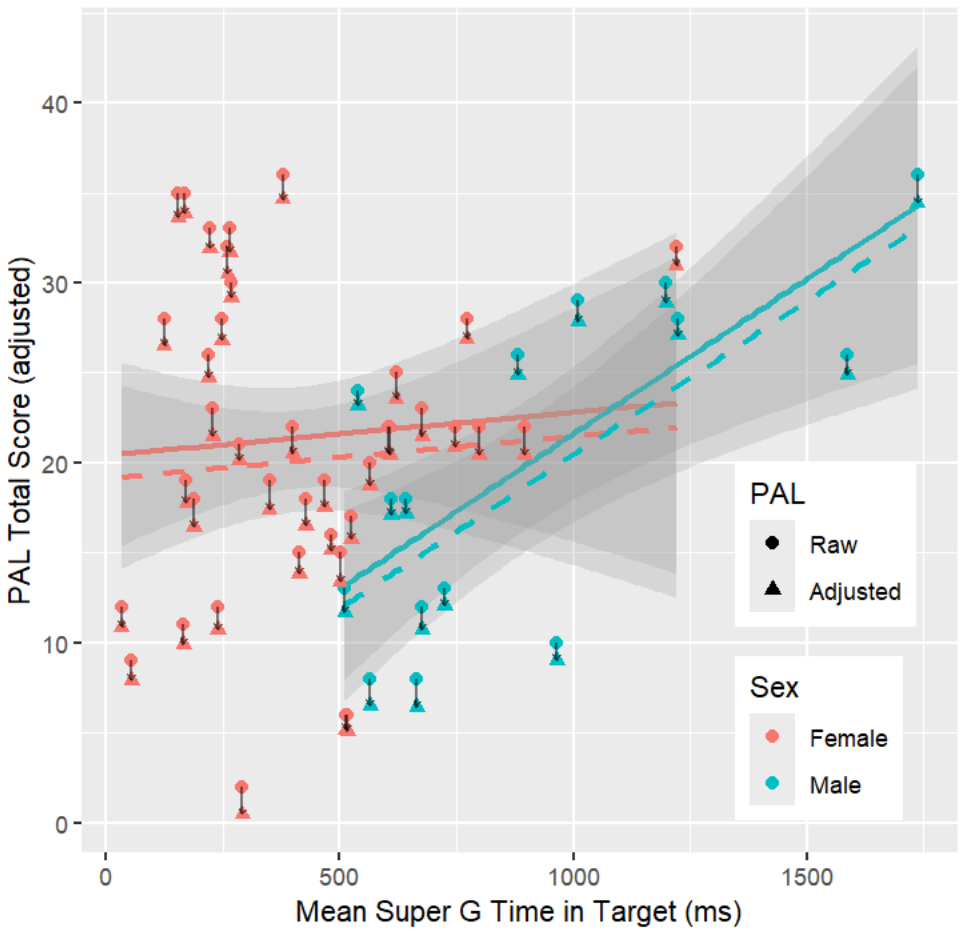

Fig 5S. The relationship between mean Super G time in target to individual PAL total score before (Raw – circle) and after (Adjusted – triangle) statistical adjustment for time between PAL measurement and Super G measurement stratified by sex (Male in Blue and Female in Pink). Given known sex differences between Males and Females on PAL and observed sex differences on mean Super time in target, such a stratification by sex was necessary to control for potential confounding of sex on the Super G to PAL relationship. The solid blue and pink lines represent the line of least squares for Raw PAL for males and females, respectively. The dashed blue and pink lines represent the line of least squares for Adjusted PAL for males and females, respectively. The gray ribbon about each line represents the 95% confidence interval.
